# Horizontal Bone Augmentation with Simultaneous Implant Placement in the Aesthetic Region: A Case Report and Review of the Current Evidence

**DOI:** 10.3390/medicina60111786

**Published:** 2024-11-01

**Authors:** Izzetti Rossana, Cinquni Chiara, Alfonsi Fortunato, Nisi Marco, Covelli Michele, Garcia Mira Berta, Priami Mattia, Barone Antonio

**Affiliations:** 1 Department of Surgical, Medical, Molecular Pathology and of the Critical Area, University of Pisa, 56126 Pisa, Italy; rossana.izzetti@unipi.it (I.R.); priami.mattia@gmail.com (P.M.); 2 Dental Biomaterials Research Unit, University of Liege, 4020 Liege, Belgium; 3 Department of Medicine and Surgery, LUM University, 70010 Bari, Italy; covelli@lum.it; 4 Department of Stomatology, University of Valencia, 46010 Valencia, Spain

**Keywords:** guided bone regeneration, dental implants, biomaterials

## Abstract

This study aimed to describe a case of simultaneous guided bone regeneration (GBR) and implant placement in a patient with prior aesthetic implant failure, focusing on achieving optimal aesthetic and functional outcomes, and to perform a literature review of the current evidence. A 38-year-old male presented with an improperly positioned implant at the level of the right upper central (tooth 2.1), leading to aesthetic and functional issues. The initial assessment included a panoramic radiograph revealing marginal bone loss and an enlarged peri-implant space, necessitating implant removal. Following implant removal and provisional restoration, the patient was re-evaluated for subsequent therapeutic treatments. The patient underwent orthodontic treatment to improve mesio-distal spacing, followed by surgical intervention involving GBR and simultaneous implant placement. The GBR procedure utilised bone substitutes and resorbable membranes, with soft tissue augmentation conducted subsequently. The surgical intervention involved local anaesthesia, flap design, defect assessment, and palatally oriented implant placement. GBR was then performed. After six months, implant uncovering and soft tissue augmentation were conducted. The implant was loaded with a screwed restoration following complete hard and soft tissue healing. The patient was monitored every six months for two years, then annually. At the 10-year follow-up, no signs of bone resorption or soft tissue inflammation were observed. This case demonstrated that GBR and simultaneous implant placement, with the application of advanced biomaterials, effectively promoted osseointegration and maintained aesthetic and functional stability over a decade.

## 1. Introduction

The pursuit of optimal aesthetic outcomes in implant-prosthetic rehabilitations has driven significant advancements in surgical techniques and biomaterials. Guided bone regeneration (GBR) has emerged as a cornerstone approach for addressing alveolar bone deficiencies prior or contextually to implant placement [1], with reproducible results [2,3].

GBR involves the application of bone substitutes [4] in association with barrier membranes to selectively exclude non-osteogenic cells, allowing bone formation while preventing soft tissue ingrowth [5]. Membrane materials have been extensively studied and refined to enhance biocompatibility, stability, and clinical efficacy [6]. Furthermore, the integration of growth factors to promote osteogenesis and angiogenesis has shown promising results in improving bone regeneration and accelerating healing processes [7]. As compared to the posterior maxillary and mandibular sites, the anterior aesthetic region presents unique challenges due to its high visibility and patient expectations regarding the final aesthetic outcome [8]. Therefore, GBR may facilitate the predictability of bone volume and architecture, essential for achieving optimal prosthetic-guided implant placement and desirable aesthetic results. Within the aesthetic zone, GBR techniques have garnered significant attention in contemporary implant dentistry. Achieving optimal bone volume and architecture in the aesthetic zone is crucial for ensuring long-term stability and harmonious integration of dental implants and restorations with surrounding soft tissues [9,10].

The aim of the present report was to describe a case of implant placement simultaneously with horizontal GBR procedures in the aesthetic region, and to perform a literature review of the current evidence.

## 2. Case Report

A 38-year-old male patient was referred for the presence of an implant placed at the level of the right upper central incisor (tooth 2.1) and a prosthetic rehabilitation with unfavourable aesthetic outcomes. Clinical examination revealed asymmetry of the gingival margin, the absence of keratinized tissue, and the presence of peri-implant probing that exceeded the physiological values (peri-implant probing ≤ 5.0 mm according to the criteria stated by the World Workshop on the Classification of Periodontal and Peri-Implant Diseases and Conditions) [11]. The implant position did not allow for proper prosthetic rehabilitation, which was splinted to the adjacent teeth. The patient required corrective treatment to address both the aesthetic and functional concerns. The patient did not report any systemic pathology nor medication intake and he was non-smoker.

The bidimensional X-ray examination showed peri-implant bone loss and enlargement of the peri-implant space. All the clinical and radiographic data suggested that implant removal was deemed appropriate.

**Figure 1:**
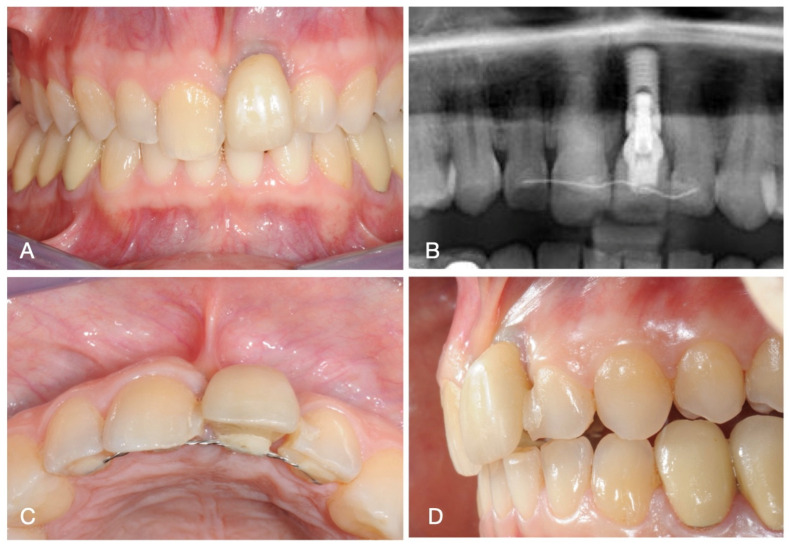
Clinical and radiographic images of initial situation. (**A**) Frontal view, (**B**) radiographic image, (**C**) occlusal view, and (**D**) lateral view.

The initial clinical and radiographic situation is presented in Figure 1.

**Figure 2:**
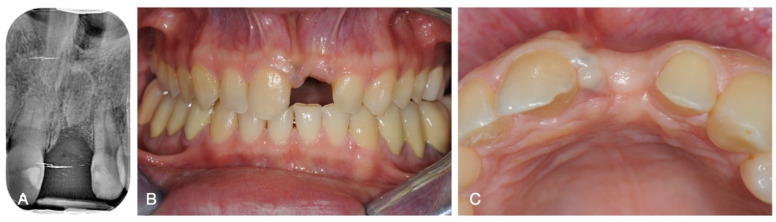
(**A**) Radiographic appearance after 3 months of healing, (**B**) frontal view, and (**C**) occlusal view.

After disassembling the crown and the implant abutment, the implant was removed with trephine burs, trying to minimise the trauma to the bony walls. Provisional restoration was performed with an essix retainer with resin bonded tooth 2.1. At three months, the healing of the implant site was evaluated, and defective width and height of the 2.1 site were observed (Figure 2).

The patient then underwent orthodontic treatment with aligners to improve the mesio-distal spacing at the level of the 2.1 site. After 10 months of orthodontic treatment, surgical intervention of the implant placement was planned. The intervention involved a first surgical procedure with GBR and simultaneous implant positioning, and a second surgery for the management of soft tissues.

**Figure 3:**
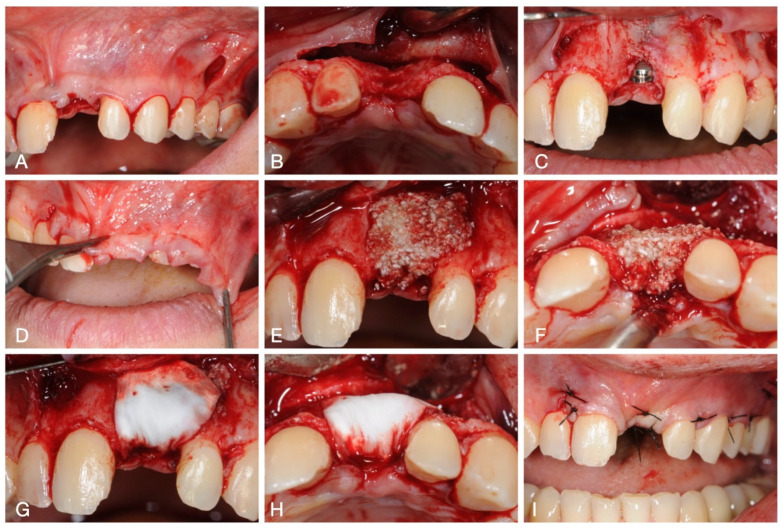
Surgical procedures: (**A**) flap incision; (**B**) flap elevation by occlusal view; (**C**) implant placement; (**D**) flap released, (**E**) biomaterial placement by frontal view, (**F**) and by occlusal view; (**G**) membrane placement from frontal view, (**H**) and from occlusal view; and (**I**) flap repositioning and sutures placement.

For the first surgical procedure, local anaesthesia with mepivacaine 2% with adrenaline 1:100,000 was administered buccally and palatally in the anterior maxillary region. A full-thickness flap was elevated from tooth 1.2 to tooth 2.4. The flap had a triangular design with distal releasing incision at the level of tooth 2.4. After raising the flap, a buccal defect was noted at the level of site 2.1. The implant receiving site was prepared according to prosthetic implant planning to achieve an adequate emergence for the final restoration. A bone-level implant (Intra-Lock, Boca Raton, FL, USA) was positioned. The flap was then mobilised through periosteal releasing incisions, and GBR was performed using a cortico-cancellous bone mix (Gen-Os, Tecnoss Dental Srl, Giaveno, Torino, Italy) combined with heterologous Type I and III collagen gel plus a thermogelling synthetic copolymer (TSV gel, Tecnoss Dental Srl, Giaveno, Torino, Italy), and a resorbable collagen membrane (Osteobiol Evolution, Tecnoss Dental Srl, Giaveno, Torino, Italy) was placed to protect the biomaterial. The flap was then repositioned after proper passivation with 4.0 non-resorbable thread (Polimid, Sweden & Martina, Due Carrare, Padova, Italy) with mattress and single sutures. All of the surgical procedures are shown in Figure 3.

The site was left to heal for six months. Subsequently, the second stage of surgery was planned. Soft tissue augmentation was performed to increase the width and thickness of the keratinized peri-implant tissue. A split-thickness flap was elevated from element 1.1 to 2.2, and a connective tissue graft (CTG) of approximately 10 × 5 mm was harvested from the palate (3 mm from the gingival margin of the elements 2.5–2.7) and positioned at site 2.1. Then, the healing abutment was applied to the implant, and the flap was apically repositioned with 5.0 resorbable sutures (Vicryl, Ethicon, Raritan, NJ, USA). The soft tissues healed for 2 months.

At this timepoint, the implant was restored with a provisional crown, as complete healing of both hard and soft tissues was observed. After 5 months of soft tissue healing, a final zirconia crown was delivered.

**Figure 4:**
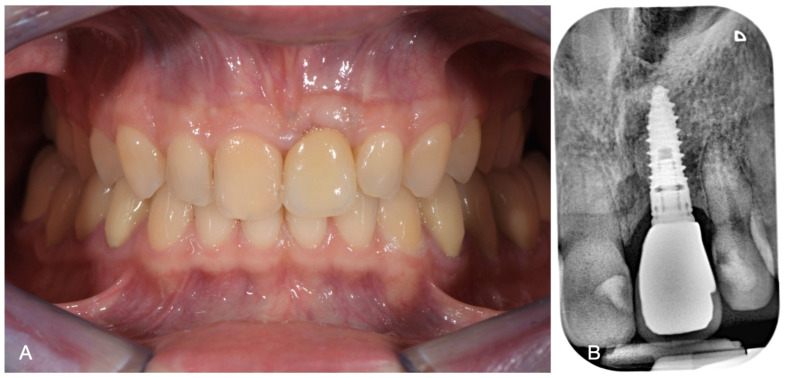
(**A**) Frontal view at follow-up and (**B**) radiographic features.

The patient entered follow-up every 6 months for the first 2 years, then recall was scheduled once a year. At the 10-year follow-up, no signs of bone resorption or soft tissue inflammation were noted, and the patient was fully satisfied both by the aesthetic and functional result. The final clinical situation after 10 years is shown in Figure 4.

## 3. Discussion

The present case report described a horizontal GBR procedure performed simultaneously to implant placement in a spontaneously healed site after previous implant removal.

Simultaneous implant placement with GBR is a widely accepted technique to manage implant placement in areas with insufficient bone volume. The aim of GBR is to enhance bone volume, ensure the stability of the implant, and improve the aesthetic outcomes [12]. In the literature, several studies investigated the long-term performance and success rates of implants placed with GBR, comparing different materials and techniques [13].

As reported in the work by Cucchi et al. [12], patient selection, defect analysis, adequate blood supply, rigorous fixation of membranes, and proper soft-tissue management are needed to avoid complications and optimise outcomes following GBR procedures. Among the suggested practises, the combined use of autogenous bone and xenograft or allograft, the removal of non-resorbable membranes after 6 to 9 months, and careful flap passivation to ensure tension-free closure are advised to achieve proper healing.

Bone volume stability and critical bone graft thickness appear extremely relevant in GBR simultaneous to implant placement in the anterior maxilla, as a significant alveolar crest collapse can be observed predominantly at the buccal aspect of the implants. As reported by Zhou et al. [14], the buccal aspect of the maxillary alveolar bone is subjected to significant and rapid resorption in the first 6 months following implant placement. Specifically, more pronounced bone reduction occurs at the implant platform level, which may be attributed to membrane collapse following flap suturing and graft displacement due to compressive forces in the augmented area [15]. A buccal bone thickness of 4.1–4.5 mm at the implant platform is considered adequate to ensure a post-healing thickness of 1.8 to 2.0 mm, which is necessary for both aesthetic and functional success [16]. According to the literature [17,18], the mean horizontal bone gain for GBR procedures performed with xenografts and resorbable membranes may vary from 3.0 to 5.6 mm. Indeed, it should be borne in mind that defects larger than 3 mm, extraosseous defects, or cases where at least 6 mm of width augmentation is required may benefit from a staged bone augmentation procedure. Conversely, smaller defects (ranging from 1 to 3 mm) and certain more extensive defects (4–5 mm) are often suitable for simultaneous augmentation methods [17]. Assessing the need for additional bone grafting to enable optimal, prosthetically guided implant placement is a critical factor in evaluating the clinical outcomes of lateral bone augmentation procedures. Adequate bone volume for precise implant positioning can often be achieved without requiring further grafting.

In terms of implant survival rate, positive outcomes of GBR have been reported across the literature. Wessels et al. (2020) [19] observed that early implant placement with GBR resulted in a 100% implant survival rate after 5 years, with no cases of peri-implantitis and a low incidence of peri-implant mucositis; the authors hypothesised the need for additional soft tissue augmentation following GBR to improve aesthetic results. Liu et al. (2019) [20] performed GBR and simultaneous implant placement, and reported 100% implant survival, irrespective of the performance of submerged versus non-submerged healing. Satisfactory results were obtained in terms of soft tissue texture, and patient satisfaction in 87% of cases. However, alveolar process deficiency was reported as the most unfavourable outcome at the 1-year follow-up. Jung et al. (2021) [21] reported a long-term survival rate of 89.3% to 93.8% for implants placed with GBR using resorbable and non-resorbable membranes over a period of 22–24 years. The authors observed the presence of stable marginal bone level, whilst the performance of GBR did not affect the amount of keratinized mucosa, buccal marginal mucosa level, and buccal bone level. In cases of thin periodontal phenotype and high aesthetic demands, it is advisable to consider placing a connective tissue graft below the marginal soft tissue level on the buccal aspect to maintain mid-facial soft tissue stability [22].

Smoking was identified as a significant negative factor affecting implant survival, as early GBR complications and a higher rate of implant loss were observed in smoker patients, therefore proper patient selection is a critical factor for successful GBR procedures. Non-smoker patients, with good oral hygiene and periodontal health status are more likely to experience favourable outcomes [19].

The case described in the present report was treated with an autogenous CTG harvested from the palate at the time of implant uncovering. The choice of using a CTG was based on its ability to increase mucosal thickness and KMW [23], and to integrate with the soft tissue colour of adjacent teeth. Moreover, a free gingival graft (FGG) could have resulted in an increased KMW, but with a higher risk of colour mismatching [24].

On the long term, soft tissue aesthetics were satisfying, suggesting that the performance of surgical procedures on soft tissues may improve aesthetic outcomes as well as reinforce the barrier role of keratinized gingival tissues.

It should be noted that some authors [25,26] suggest that in cases of smaller defects (<2–3 mm), bone filling could also be achieved in the absence of additional grafting procedures. For instance, Block and Kent [27] reported a 100% implant success rate in small, non-grafted defects, compared to a 93% success rate in larger, grafted defects. Similarly, Becker et al. [28] reported a 93% success rate in smaller defects, compared with 76–83% in larger defects needing grafting. A systematic review by AlKudami et al. [29] highlighted the presence of a significant advantage in performing GBR procedures for preserving buccal-palatal ridge dimensions and buccal bone thickness, irrespective of the variations in surgical techniques. Importantly, such procedures also reflect positive outcomes in soft tissue height and thickness. Nevertheless, while GBR performance with simultaneous implant placement may favour horizontal ridge preservation, such procedures find indication in the presence of gaps > 2 mm, as in the adequate bone fill of smaller defects has been observed to occur with or without GBR.

## 4. Conclusions

Simultaneous implant placement with GBR is nowadays a widely accepted technique to manage implant placement in areas with insufficient bone volume. The present case report described a horizontal GBR procedure performed simultaneously to implant placement in a spontaneously healed site after previous implant removal. According to the literature, GBR with simultaneous implant placement appears to be a reliable technique for managing cases with insufficient bone volume, providing high survival rates and satisfactory aesthetic outcomes. However, additional soft tissue augmentation may be required to optimise aesthetic results, particularly in aesthetically demanding cases. Indeed, the case described in the present report was treated with an autogenous connective tissue graft harvested from the palate at the time of implant uncovering. On the long term, soft tissue aesthetics were satisfying, suggesting that the performance of surgical procedures on soft tissues may improve aesthetic outcomes as well as reinforce the barrier role of keratinized gingival tissues. Clinicians should tailor their approach based on individual patient needs and clinical conditions to achieve the best functional and aesthetic outcomes.

## Data Availability

Data are contained within the article.
